# Dissemination and Stability of the *bla*_NDM-5_-Carrying IncX3-Type Plasmid among Multiclonal Klebsiella pneumoniae Isolates

**DOI:** 10.1128/mSphere.00917-20

**Published:** 2020-11-04

**Authors:** Wenjun Zhu, Xing Wang, Juanxiu Qin, Wei Liang, Zhen Shen

**Affiliations:** a Department of Laboratory Medicine, the Second People's Hospital of Lianyungang City, Lianyungang, Jiangsu Province, China; b Department of Laboratory Medicine, Shanghai Children's Medical Center, School of Medicine, Shanghai Jiao Tong University, Shanghai, China; c Department of Laboratory Medicine, Renji Hospital, School of Medicine, Shanghai Jiaotong University, Shanghai, China; Escola Paulista de Medicina/Universidade Federal de São Paulo

**Keywords:** IncX3 plasmid, *K. pneumoniae*, NDM-5, conjugal transfer, plasmid stability

## Abstract

NDM-5 carbapenemase was mainly identified in Escherichia coli, while the rapid transmission of *bla*_NDM-5_ among *Enterobacteriaceae* has raised serious public attention. This study identified 14 NDM-5-producing Klebsiella pneumoniae isolates from 107 carbapenem-resistant K. pneumoniae isolates, recovered from blood, urine, and normally sterile body fluids of pediatric patients from January 2016 to December 2018. All NDM-5-producing isolates were highly resistant to β-lactams, while tigecycline and polymyxin B exhibited excellent antimicrobial activity. These 14 strains belonged to 9 different sequence types (STs) and displayed various pulsed-field gel electrophoresis (PFGE) patterns, suggesting that they were not clonally related. S1-PFGE followed by Southern blotting showed that the *bla*_NDM-5_ gene was located on an ∼46-kb IncX3 plasmid in all strains. All *bla*_NDM-5_-carrying plasmids were successfully transferred into recipient E. coli J53. PCR-based sequencing demonstrated that all of the *bla*_NDM-5_-carrying plasmids shared highly similar backbones, with nucleotide sequence identity of >99%. Moreover, this plasmid displayed high sequence similarity to the previously reported epidemic IncX3 *bla*_NDM-5_-carrying plasmids, with dynamic changes observed only in *bla*_NDM-5_-surrounding elements. Interestingly, the IncX3 *bla*_NDM-5_-carrying plasmids showed strong stability in clinical isolates when cultured in antibiotic-free medium. However, after the conjugation inhibitor linoleic acid was added, a gradual increase in the level of IncX3 plasmid loss could be observed. Clinical isolates displayed 10% to 15% *bla*_NDM-5_-carrying plasmid loss after coculture with linoleic acid for 5 days. These results showed that the IncX3 plasmid facilitated the dissemination of *bla*_NDM-5_ among multiclonal K. pneumoniae strains in children and that conjugal transfer contributed significantly to IncX3 plasmid stability within K. pneumoniae.

**IMPORTANCE** The emergence and spread of New Delhi metallo-β-lactamase (NDM)-producing *Enterobacteriaceae* have been a serious challenge to public health, and NDM-5 shows increased resistance to carbapenems compared with other variants. NDM-5 has been identified mostly in E. coli but has rarely been described in K. pneumoniae and other *Enterobacteriaceae* isolates. Here, we present the dissemination of highly similar 46-kb IncX3 *bla*_NDM-5_-carrying plasmids among multiclonal K. pneumoniae strains in children, highlighting the horizontal gene transfer of *bla*_NDM-5_ among K. pneumoniae strains via the IncX3 plasmid. Moreover, the IncX3 *bla*_NDM-5_-carrying plasmids displayed strong stability in clinical strains when cultured in antibiotic-free medium, and the plasmid maintenance was attributed partly to conjugal transfer. Plasmid conjugation is mediated by the type IV secretion system (T4SS), and T4SS is conserved among all epidemic IncX3 *bla*_NDM-5_-carrying plasmids. Therefore, combining conjugation inhibition and promotion of plasmid loss would be an effective strategy to limit the conjugation-assisted persistence of IncX3 *bla*_NDM-5_-carrying plasmids.

## Abstract (precis)

The emergence and spread of New Delhi metallo-β-lactamase (NDM)-producing *Enterobacteriaceae* have been a serious challenge to public health, and NDM-5 shows increased resistance to carbapenems compared with other variants. NDM-5 has been identified mostly in E. coli but has rarely been described in K. pneumoniae and other *Enterobacteriaceae* isolates. Here, we present the dissemination of highly similar 46-kb IncX3 *bla*_NDM-5_-carrying plasmids among multiclonal K. pneumoniae strains in children, highlighting the horizontal gene transfer of *bla*_NDM-5_ among K. pneumoniae strains via the IncX3 plasmid. Moreover, the IncX3 *bla*_NDM-5_-carrying plasmids displayed strong stability in clinical strains when cultured in antibiotic-free medium, and the plasmid maintenance was attributed partly to conjugal transfer. Plasmid conjugation is mediated by the type IV secretion system (T4SS), and T4SS is conserved among all epidemic IncX3 *bla*_NDM-5_-carrying plasmids. Therefore, combining conjugation inhibition and promotion of plasmid loss would be an effective strategy to limit the conjugation-assisted persistence of IncX3 *bla*_NDM-5_-carrying plasmids.

## OBSERVATION

NDM (New Delhi metallo-β-lactamase) carbapenemase is an important type of carbapenemase with the ability to hydrolyze almost all β-lactams, and 24 NDM variants (NDM-1 to NDM-24) have been identified to date (1). The NDM-5 carbapenemase differs from NDM-1 by only two amino acid substitutions (Val88Leu and Met154Leu) and shows increased resistance to carbapenems and expanded-spectrum cephalosporins (2). NDM-5 has been reported all over the world, but it has been identified mainly in Escherichia coli (3, 4). Recently, clonal dissemination of NDM-5-producing Klebsiella pneumoniae strains was reported in children (5), suggesting the rapid transmission of *bla*_NDM-5_ among *Enterobacteriaceae*. Here, we present the dissemination of the IncX3 *bla*_NDM-5_-carrying plasmid among multiclonal K. pneumoniae strains in children, and the stability of IncX3 *bla*_NDM-5_-carrying plasmid within K. pneumoniae was also investigated.

## 

### Dissemination of *BLA*_NDM-5_ among multiclonal K. pneumoniae strains in children.

A total of 107 clinical isolates of carbapenem-resistant K. pneumoniae (CRKP) were recovered from blood, urine, and normally sterile body fluids (SBF) of patients in Shanghai Children’s Medical Center from January 2016 to December 2018. All isolates were analyzed by PCR for *bla*_KPC_, as well as *bla*_NDM,_ *bla*_IMP,_ *bla*_OXA-48_, and *bla*_VIM_ (6); 65 *bla*_NDM-1_-positive, 28 *bla*_KPC-2_-positive, and 14 *bla*_NDM-5_-positive isolates were identified. Antimicrobial susceptibility was determined for all *bla*_NDM-5_-positive isolates, using the broth microdilution method according to the guidelines of Clinical and Laboratory Standards Institute (CLSI) (7). NDM-5-producing K. pneumoniae was highly resistant to all β-lactams tested, with the exception of aztreonam (Table 1). PCR detection of extended-spectrum β-lactamase (ESBL) genes (*bla*_CTX-M-1_, *bla*_CTX-M-2_, *bla*_CTX-M-8/25_, *bla*_CTX-M-9_, *bla*_SHV_, and *bla*_TEM_) was performed for the selected NDM-5-positive isolates (8). Except for 4 isolates, all showed positive detection of ESBL genes. Among other antimicrobial agents, amikacin, levofloxacin, tigecycline, and polymyxin B exhibited excellent activity against these isolates. Furthermore, NDM-5-producing K. pneumoniae strains belonged to 9 different sequence types (STs) (Table 1). In accordance with the multilocus sequence type (MLST) results, these strains displayed various pulsed-field gel electrophoresis (PFGE) patterns (see Fig. S1 in the supplemental material).

**TABLE 1:** Antimicrobial susceptibility of NDM-5-producing K. pneumoniae

Strain	ST	Specimen	β-Lactamase(s)^*a*^	MIC (μg/ml)^*b*^								
				MEM	IPM	FEP	ATM	CAZ	AMK	LVX	TGC	POL
K2-1	37	Blood	NDM-5, CTX-M-14, SHV-12	64	32	128	>128	>128	0.5	0.5	≤0.25	0.5
K2-3	37	Blood	NDM-5	64	16	32	1	>128	0.5	≤0.25	≤0.25	1
K2-4	37	Blood	NDM-5, CTX-M-14, SHV-12	64	16	128	>128	>128	0.5	0.5	≤0.25	0.5
K2-6	659	Urine	NDM-5	>128	>128	>128	0.5	>128	1	≤0.25	≤0.25	0.5
K2-7	48	Blood	NDM-5, CTX-M-15	128	64	>128	64	>128	1	0.5	≤0.25	0.5
K3-4	111	Blood	NDM-5	128	32	128	≤0.25	>128	0.5	≤0.25	≤0.25	1
K4-2	307	Ascites	NDM-5, CTX-M-15	64	16	64	64	>128	1	1	≤0.25	1
K4-6	48	Urine	NDM-5, CTX-M-3	128	128	>128	>128	>128	0.5	0.5	≤0.25	≤0.25
K4-7	656	Blood	NDM-5, CTX-M-14	>128	>128	>128	16	>128	1	4	≤0.25	2
K6-2	785	Blood	NDM-5, CTX-M-65	128	64	>128	32	>128	>128	≤0.25	≤0.25	0.5
K6-6	2033	Blood	NDM-5, CTX-M-15	64	16	128	128	>128	1	2	0.5	0.5
K6-7	785	Urine	NDM-5, CTX-M-65	128	64	128	64	>128	>128	≤0.25	≤0.25	0.5
K6-8	307	Blood	NDM-5, CTX-M-15	64	16	128	64	>128	1	1	≤0.25	≤0.25
K7-7	824	Urine	NDM-5	64	16	32	≤0.25	>128	1	≤0.25	≤0.25	0.5

a Only NDM-5 and extended-spectrum-β-lactamase (ESBL) enzymes are listed.

b MEM, meropenem; IPM, imipenem; FEP, cefepime; ATM, aztreonam; CAZ, ceftazidime; AMK, amikacin; LVX, levofloxacin; TGC, tigecycline; POL, polymyxin B.

10.1128/mSphere.00917-20.1 FIG S1 PFGE analysis of the representative NDM-5-producing K. pneumoniae strains. Numbers represent the strain codes. M, DNA marker. Download FIG S1, JPG file, 0.3 MB. Copyright © 2020 Zhu et al. 2020 Zhu et al. This content is distributed under the terms of the Creative Commons Attribution 4.0 International license.

### The IncX3 plasmid facilitated the dissemination of *BLA*_NDM-5._

S1-PFGE and Southern blot analysis demonstrated that all of the NDM-5-producing K. pneumoniae isolates possessed 2 to 3 plasmids and that the *bla*_NDM-5_ genes were all located on plasmids with similar sizes (∼46 kb) (Fig. S2). The *bla*_NDM-5_-carrying plasmids of all 14 K. pneumoniae isolates could be transferred into recipient E. coli strain J53, at a frequency of 3.5 × 10^−4^ to 6.6 × 10^−4^ transconjugants per donor cell. The transconjugants exhibited significantly increased resistance to carbapenems compared with E. coli J53 (see Table S2 in the supplemental material). All *bla*_NDM-5_-carrying plasmids were classified as IncX3 type through PCR-based replicon typing (9). The genetic relatedness among *bla*_NDM-5_-carrying plasmids from different strains was determined through PCR-based sequencing for *bla*_NDM-5_ surrounding elements and the type IV secretion system (T4SS) (10). All of the *bla*_NDM-5_-carrying plasmids shared highly similar backbones, including *bla*_NDM-5_ genetic elements and T4SS, with nucleotide sequence identity of >99%.

10.1128/mSphere.00917-20.2 FIG S2 S1-digested plasmid DNA and Southern blot hybridization for representative NDM-5-prducing K. pneumoniae strains. Bands indicated with arrows show positive signals in Southern blot hybridization with the *bla*_NDM-5_ probe. M, DNA marker. Download FIG S2, JPG file, 0.4 MB. Copyright © 2020 Zhu et al. 2020 Zhu et al. This content is distributed under the terms of the Creative Commons Attribution 4.0 International license.

10.1128/mSphere.00917-20.3 TABLE S1 Primers used for screening the backbone of *bla*_NDM-5_-carrying plasmids. Download Table S1, DOCX file, 0.02 MB. Copyright © 2020 Zhu et al. 2020 Zhu et al. This content is distributed under the terms of the Creative Commons Attribution 4.0 International license.

10.1128/mSphere.00917-20.4 TABLE S2 Antimicrobial susceptibility testing of *bla*_NDM-5_ transconjugants. Download Table S2, DOCX file, 0.02 MB. Copyright © 2020 Zhu et al. 2020 Zhu et al. This content is distributed under the terms of the Creative Commons Attribution 4.0 International license.

**FIG 1:**
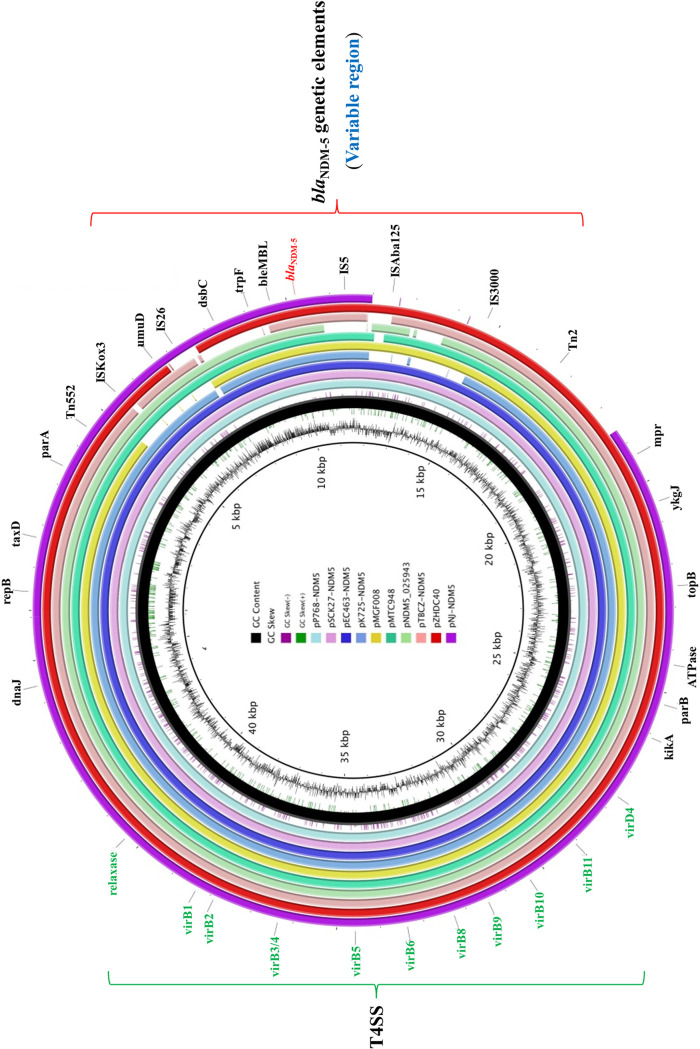
Comparative analysis of pSCK27-NDM5 with other reference IncX3 *bla*_NDM-5_-carrying plasmids. The type IV secretion system (T4SS) and *bla*_NDM-5_ genetic elements are indicated. The circular map was created by the use of the BLAST Ring Image Generator (BRIG). Concentric rings represent the similarity between pSCK27-NDM5 in the inner ring and other reference sequences in the outer rings. The nine reference IncX3 plasmid sequences were obtained from GenBank and were listed with plasmid name (GenBank accession number, bacterial host, country of detection): pP768-NDM5 (MF547510, E. coli, China), pEC463-NDM5 (MG545911, E. coli, China), pK725-NDM5 (MK450348, K. pneumoniae, China), pMGF008 (NEWC01000014, *K. quasipneumoniae*, Malaysia), pMTC948 (MH349095, E. coli, China), pNDM5_025943 (CP027204, E. coli, China), pTBCZ-NDM5 (MH107030, K. pneumoniae, China), pZHDC40 (KY041843, E. coli, China) and pNJ-NDM5 (KX447767, E. coli, United States).

K. pneumoniae strain K2-7 was randomly selected, and plasmid DNA (pSCK27-NDM5) from the corresponding E. coli J53 transconjugant was extracted using a Qiagen Plasmid midi kit (Qiagen, Hilden, Germany) and was sequenced with an Illumina MiSeq system (Illumina, CA, USA). The reads were assembled *de novo* into contigs using SPAdes 3.9.0, and gaps were closed through PCR and Sanger sequencing. Comparative analysis of the representative fully sequenced IncX3 *bla*_NDM-5_-carrying plasmids was performed to assess the genetic context of the *bla*_NDM-5_ gene. The functional genes were identical across IncX3 *bla*_NDM-5_-carrying plasmids, with all plasmids carrying genes for replication (*repB*), partitioning (*parA* and *parB*), and conjugative transfer (*virB1*, *virB2*, *virB3*/*4*, *virB5*, *virB6*, *virB8*, *virB9*, *virB10*, *virB11*, and *virD4*) (Fig. 1). Structural differences resulting from potential insertions or deletions were observed only on *bla*_NDM-5_ genetic surrounding elements, which could also be regarded as the variable region of the IncX3 plasmids. The variable region on IncX3 plasmids is highly dynamic, and it is unclear if these differences have any effect on the expression of *bla*_NDM-5_.

### Conjugal transfer contributed significantly to IncX3 plasmid stability within K. pneumoniae.

The stability of the IncX3 *bla*_NDM-5_-carrying plasmid within K. pneumoniae was investigated, and three NDM-5-producing strains (K2-7, K4-2, and K6-2) were randomly selected. The proportion of the bacterial population that retained the *bla*_NDM-5_-carrying plasmid was determined over a period of 5 days (11). Bacteria were subcultured into antibiotic-free Luria-Bertani (LB) broth at a dilution of 1 in 1,000 daily. In order to investigate the impact of conjugal transfer on the stability of *bla*_NDM-5_-carrying plasmid, the conjugation inhibitor linoleic acid was added to LB broth at final concentrations of 2.5 and 5 mM. The culture was diluted each day, and each dilution was plated on LB agar and incubated overnight at 37°C. A total of 100 colonies were randomly collected from all dilutions and spotted on LB plates in the presence and absence of meropenem. Plasmid retention was calculated by comparing the number of colonies on the LB agar plate containing meropenem with that on pure LB agar.

**FIG 2:**
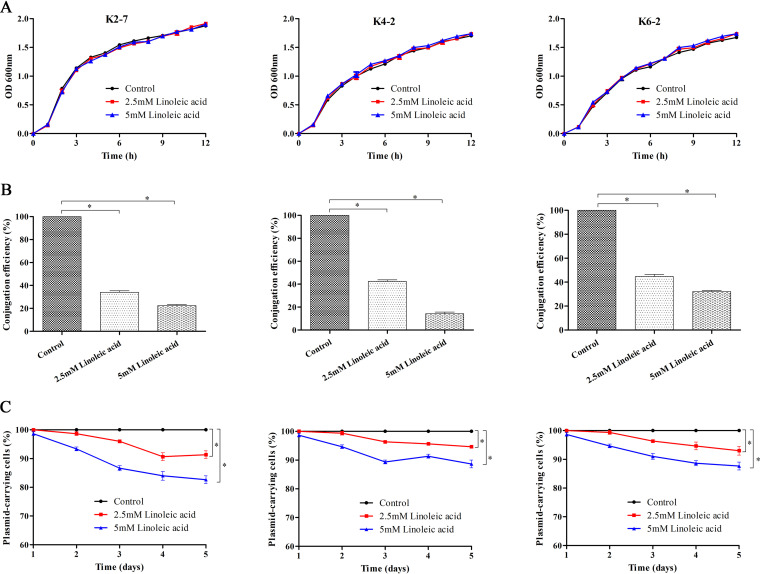
IncX3 *bla*_NDM-5_ plasmid loss assay in strains K2-7, K4-2, and K6-2. (A) Bacterial growth curve of strains in LB broth with or without linoleic acid. OD 600nm, optical density at 600 nm. (B) Bacterial conjugation was monitored through liquid-mating conjugation assay in the presence of 2.5 mM or 5 mM linoleic acid. “Conjugation efficiency” refers to the relative conjugation frequencies of the IncX3 *bla*_NDM-5_-carrying plasmids after adding the conjugation inhibitor linoleic acid. (C) IncX3 *bla*_NDM-5_ plasmid stability in strains cultured with or without linoleic acid. Bacteria were subcultured into fresh LB broth without antibiotics at a dilution of 1 in 1,000 daily for 5 days. The experiment was repeated on three separate occasions, and error bars represent standard deviations. *, *P* < 0.05, one-way analysis of variance (ANOVA) with Bonferroni correction.

The IncX3 *bla*_NDM-5_-carrying plasmids showed strong stability in clinical isolates, without apparent plasmid loss after serial subculture for 5 days (Fig. 2). It seems that reducing antibiotic use alone is likely insufficient for reversing resistance. However, after the conjugation inhibitor linoleic acid was added, a gradually increase in the level of *bla*_NDM-5_-carrying plasmid loss could be observed in all three strains (Fig. 2). Linoleic acid targets type IV secretion traffic ATPase VirB11, and addition of linoleic acid can significantly decrease the conjugation efficiency of several plasmid groups (12, 13). Linoleic acid significantly decreased *bla*_NDM-5_-plasmid conjugation efficiency but did not exert any tremendous effect on bacterial growth (Fig. 2). These strains displayed 10% to 15% *bla*_NDM-5_-carrying plasmid loss after coculture with linoleic acid for 5 days, indicating that conjugal transfer contributed significantly to the persistence of IncX3 *bla*_NDM-5_-carrying plasmid. Plasmid conjugation is mediated by T4SS, and T4SS is conserved among all epidemic IncX3 *bla*_NDM-5_-carrying plasmids (14). Therefore, combining conjugation inhibition and promotion of plasmid loss would be an effective strategy to limit the conjugation-assisted persistence of IncX3 *bla*_NDM-5_-carrying plasmid.

It is commonly believed that a plasmid-free bacterial host can compete successfully with bacterial cells harboring plasmids, due to the fitness costs of plasmid carriage (15). However, recent studies indicated that significant changes in chromosomal and epidemic resistance plasmid gene expression may have allowed K. pneumoniae to ameliorate the associated fitness costs of plasmid carriage (11). Though the plasmid loss assay in this study lasted for 5 days, the results do not mean that coculture of IncX3 plasmid-free and plasmid-harboring K. pneumoniae for relatively long periods would definitely lead to an increased level of plasmid loss in bacterial populations. In addition, 3% to 5% plasmid loss was still observed in clinical strains after 1 day of culture with 5 mM linoleic acid, suggesting that inhibition of conjugal transfer is likely to promote IncX3 *bla*_NDM-5_-carrying plasmid loss from K. pneumoniae.

In summary, this study presented the dissemination of highly similar 46-kb IncX3 *bla*_NDM-5_-carrying plasmids among multiclonal K. pneumoniae strains in children, highlighting the horizontal gene transfer of *bla*_NDM-5_ among K. pneumoniae via the IncX3 plasmid. Moreover, the IncX3 *bla*_NDM-5_-carrying plasmids displayed strong stability in clinical strains when cultured in antibiotic-free medium, and conjugal transfer contributed significantly to plasmid maintenance within K. pneumoniae.

All procedures in this study that involved human participants were performed in accordance with the ethical standards of the Institutional Review Board Ethics Committee of Shanghai Children's Medical Center. For this type of retrospective study, formal consent is not required.

### Data availability.

The complete sequence of plasmid pSCK27-NDM5 was submitted to the GenBank database under accession number MT663954.

10.1128/mSphere.00917-20.5 DATA SET S1 The complete plasmid sequence of pSCK27-NDM5. Download Data Set S1, TXT file, 0.04 MB. Copyright © 2020 Zhu et al. 2020 Zhu et al. This content is distributed under the terms of the Creative Commons Attribution 4.0 International license.
