# Supplementary figures and images for: Dissemination and Stability of the blaNDM-5-Carrying IncX3-Type Plasmid among Multiclonal Klebsiella pneumoniae Isolates
Source: mSphere. 2020 Nov 4;5(6):e00917-20. doi: 10.1128/mSphere.00917-20 (PMC7643832; doi:10.1128/mSphere.00917-20)

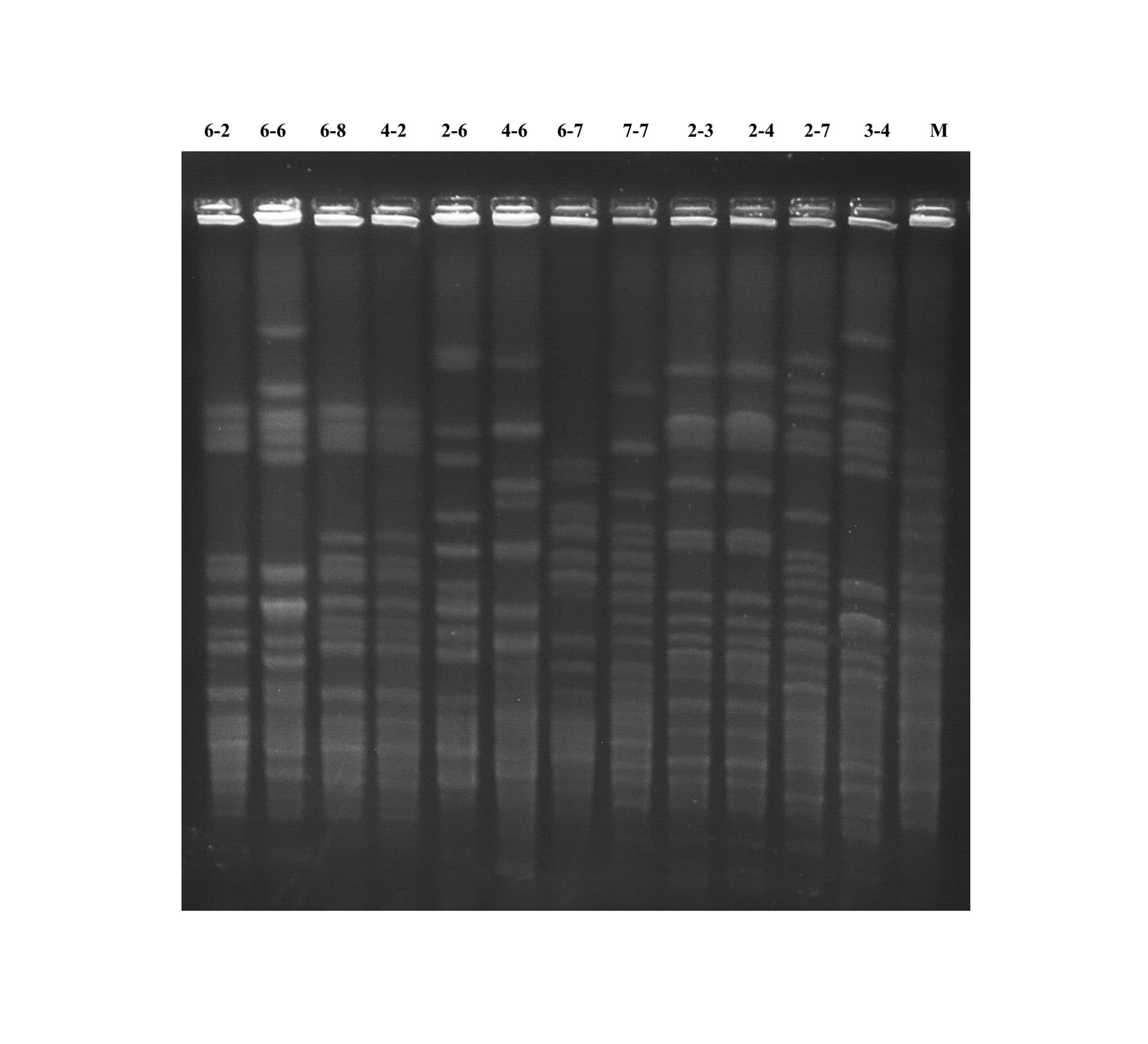

Supplement: FIG S1 [file mSphere.00917-20-sf001.jpg]

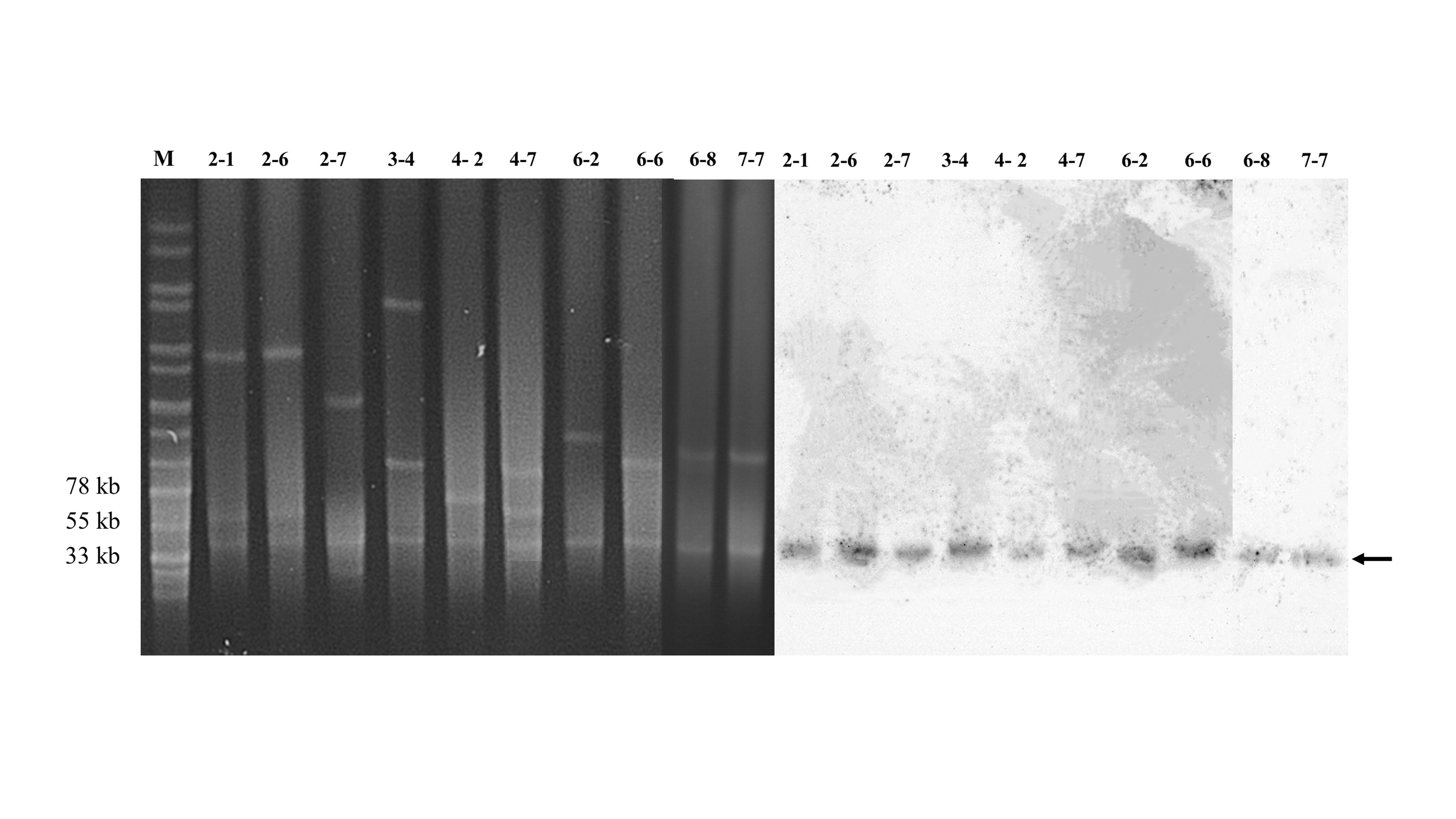

Supplement: FIG S2 [file mSphere.00917-20-sf002.jpg]
