# Supplementary material for: Dissemination and Stability of the blaNDM-5-Carrying IncX3-Type Plasmid among Multiclonal Klebsiella pneumoniae Isolates
Source: mSphere. 2020 Nov 4;5(6):e00917-20. doi: 10.1128/mSphere.00917-20 (PMC7643832; doi:10.1128/mSphere.00917-20)
Supplement: TABLE S1 [file mSphere.00917-20-st001.docx]

Table S1. Primers used for screening the backbone of *bla*_NDM-5_-carrying plasmids

| Primers | Sequence (5'-3') | Target (bp) | Reference |
| --- | --- | --- | --- |
| umuD_F | ATGCGATTGCGACTACAC | 1460 | 1 |
| dsbC_R | TGGTACTTCACGGTCAGG |  |  |
| bleo_F | TGGGTCGAGGTCAGGATAGG | 1070 | 1 |
| IS5_R | GCGATTGATGGTCTTGAAC |  |  |
| ISAba125_F | TGAGTCAGCCGAGAAGAA | 742 | 1 |
| IS3000_R | CCAAGGAGATACCAAGAGAAT |  |  |
| vriB2_F | ATGGCGCAACAATAAAGATG | 2083 | This study |
| relaxase_R | AAGAAACAGCAACTCCAGAT |  |  |
| virB6_F | GCCTGAGTTGCACTATCTAA | 1510 | This study |
| virB4_R | GAAGGGGGAAAGTGATGATT |  |  |
| virB10_F | CACGAATAATCGACGGGATA | 1488 | This study |
| virB8_R | CTTTAATCAACGGCAACCAG |  |  |
| virD4_F | TCTGTTATTATCCGCCACAG | 730 | This study |
| virB11_R | GAATGCCTGGTTAAGGGTAA |  |  |

**REFERENCES**

1. Zhang Q, Lv L, Huang X, Huang Y, Zhuang Z, Lu J, Liu E, Wan M, Xun H, Zhang Z, Huang J, Song Q, Zhuo C, Liu JH. 2019. Rapid Increase in carbapenemase-Producing *Enterobacteriaceae* in Retail Meat Driven by the Spread of the *bla*_NDM-5_-Carrying IncX3 Plasmid in China from 2016 to 2018. Antimicrob Agents Chemother 63.
