# Supplementary material for: Dissemination and Stability of the blaNDM-5-Carrying IncX3-Type Plasmid among Multiclonal Klebsiella pneumoniae Isolates
Source: mSphere. 2020 Nov 4;5(6):e00917-20. doi: 10.1128/mSphere.00917-20 (PMC7643832; doi:10.1128/mSphere.00917-20)
Supplement: TABLE S2 [file mSphere.00917-20-st002.docx]

Table S2. Antimicrobial susceptibility testing of *bla*_NDM-5_ transconjugants

| Transconjugants | MIC (μg/mL) | | | | | | | | |
| --- | --- | --- | --- | --- | --- | --- | --- | --- | --- |
|  | IPM | MEM | FEP | ATM | CAZ | AMK | LVX | TGC | POL |
| TC-K2-1 | 8 | 16 | >32 | ≤0.5 | >32 | 1 | ≤0.25 | ≤0.25 | ≤0.25 |
| TC-K2-3 | 8 | 16 | >32 | ≤0.5 | >32 | 1 | ≤0.25 | ≤0.25 | ≤0.25 |
| TC-K2-4 | 16 | 16 | >32 | ≤0.5 | >32 | 1 | ≤0.25 | ≤0.25 | ≤0.25 |
| TC-K2-6 | 8 | 32 | >32 | ≤0.5 | >32 | 1 | ≤0.25 | ≤0.25 | ≤0.25 |
| TC-K2-7 | 16 | 32 | >32 | ≤0.5 | >32 | 1 | ≤0.25 | ≤0.25 | ≤0.25 |
| TC-K3-4 | 8 | 16 | >32 | ≤0.5 | >32 | 1 | ≤0.25 | ≤0.25 | ≤0.25 |
| TC-K4-2 | 8 | 32 | >32 | ≤0.5 | >32 | 0.5 | ≤0.25 | ≤0.25 | ≤0.25 |
| TC-K4-6 | 16 | 16 | >32 | ≤0.5 | >32 | 1 | ≤0.25 | ≤0.25 | ≤0.25 |
| TC-K4-7 | 8 | 16 | >32 | ≤0.5 | >32 | 1 | ≤0.25 | ≤0.25 | ≤0.25 |
| TC-K6-2 | 16 | 16 | >32 | ≤0.5 | >32 | 1 | ≤0.25 | ≤0.25 | ≤0.25 |
| TC-K6-6 | 8 | 16 | >32 | ≤0.5 | >32 | 1 | ≤0.25 | ≤0.25 | ≤0.25 |
| TC-K6-7 | 8 | 32 | >32 | ≤0.5 | >32 | 1 | ≤0.25 | ≤0.25 | ≤0.25 |
| TC-K6-8 | 16 | 32 | >32 | ≤0.5 | >32 | 0.5 | ≤0.25 | ≤0.25 | ≤0.25 |
| TC-K7-7 | 8 | 16 | >32 | ≤0.5 | >32 | 1 | ≤0.25 | ≤0.25 | ≤0.25 |
| J53 | ≤0.25 | ≤0.25 | ≤0.25 | ≤0.5 | 0.5 | 1 | ≤0.25 | ≤0.25 | ≤0.25 |

Abbreviations: MIC, minimum inhibitory concentration; MEM, meropenem; IPM, imipenem; FEP, cefepime; ATM, aztreonam; CAZ, ceftazidime; AMK, amikacin; LVX, levofloxacin; TGC, tigecycline; POL, polymycin B.
